# *Lactobacillus plantarum*-derived cytoplasmic membrane vesicles as novel anti-inflammatory nanotherapeutics for psoriasis management

**DOI:** 10.3389/fimmu.2025.1647466

**Published:** 2025-10-08

**Authors:** Yuedong Xie, Guowen Lv, Dandan Su, Manchun Li, Quanle Xu, Hongbo Chen, Fang Cheng, Dongling Dai

**Affiliations:** ^1^ International Medical Center, Endoscopy Center and Gastroenterology Department, Shenzhen Children's Hospital, Shenzhen, China; ^2^ School of Pharmaceutical Sciences (Shenzhen), Sun Yat-sen University, Shenzhen, China; ^3^ College of Life Sciences, Northwest A&F University, Yangling, China

**Keywords:** *Lactobacillus plantarum*, bacterial membrane vesicles, psoriasis, anandamide, precision microbiome therapy

## Abstract

**Introduction:**

Current research underscores the critical role of the gut-skin axis in inflammatory skin disorders such as psoriasis, with growing interest in the therapeutic application of probiotics. Despite this promise, the specific mechanisms and bioactive compounds through which probiotics exert their effects remain poorly characterized. In this study, we aimed to systematically evaluate the therapeutic potential of *Lactobacillus plantarum* (Lp)-derived bioactive fractions in psoriasis, with a particular focus on identifying key anti-inflammatory and immunomodulatory metabolites.

**Methods:**

The microbiome characteristics of psoriasis were analyzed through open microflora data mining and bibliometrics, and the probiotics with potential therapeutic effects were identified. Four bioactive fractions from *L. plantarum* were extracted and characterized. CCK-8, qPCR, and flow cytometry were used to evaluate the effects of four bioactive components on oxidative stress in keratinocytes and inflammatory responses in macrophages. Metabolomics was used to analyze the metabolic profiles of bioactive components with anti-inflammatory and antioxidant properties, and to screen and identify the main metabolite that play a role. To evaluate the efficacy and safety of bioactive components in the treatment of IMQ-induced psoriasis in mice.

**Results:**

A common feature of downregulation of *Lactobacillus* abundance was shown in patients with four inflammatory skin diseases including psoriasis. Four bioactive fractions, namely cytoplastic membrane vesicles (CMVs), bacterial lysate supernatant (BL-S), bacterial lysate precipitate (BL-P) and cell-free fermentation supernatant (CFS), were extracted from *L. plantarum* ATCC BAA-793, and CMVs were identified as having typical extracellular vesicles. In efficacy evaluation, CMVs and BL-S significantly reduced the mRNA levels of inflammatory factors in macrophages and the ROS levels of inflamed keratinocytes, among which CMVs had a more significant anti-inflammatory effect and had a unique inhibitory effect on M1 polarization of macrophages. Metabolomics revealed significant differences in metabolite profiles between CMVs and BL-S, and AEA enriched in both played anti-inflammatory, antioxidant and inhibitory roles in macrophage M1 polarization. In IMQ-induced psoriasis mouse models, CMVs demonstrated superior effects over BL-S in anti-hyperkeratosis, inhibiting inflammatory factor production, and down-regulating the proportion of M1 macrophages in skin and spleen. Both showed good biosafety *in vivo*.

**Discussion:**

This study demonstrates that *L. plantarum*-derived CMVs, enriched with AEA, ameliorate psoriasis through multi-faceted mechanisms including anti-inflammation, antioxidative stress reduction, and reprogramming of macrophage polarization. These findings not only position bacterial nanovesicles as a novel cell-free therapeutic strategy for inflammatory skin diseases but also establish a functional screening platform for precision microbiome-based interventions.

## 1 Introduction

The intricate interplay between the human microbiota and host immunity has redefined our understanding of health and disease within the framework of as a symbiotic ecosystem (1, 2). A disrupted microbiota system is increasingly recognized as a significant contributing factor of systemic inflammation and immune dysfunction, offering critical insights into both disease mechanisms and therapeutic opportunities (3, 4). This is particularly relevant for chronic inflammatory diseases, where a disturbed microbiota, especially the beneficial symbiotic flora, may cause systemic inflammation and immune imbalance. Psoriasis, although classified as an autoimmune disease (5, 6), exemplifies the intimate association between microbiota and disease progression (7, 8). Psoriasis patients exhibit marked reductions in cutaneous and intestinal microbial diversity (9, 10), featuring depletion of immunoregulatory genera including *Lactobacillus*, *Propionibacterium*, *Burkholderia*, *Parabacteroides*, *Bifidobacterium* and *Enterococcus*, alongside pathogenic blooms of *Staphylococcus*, *Salmonella*, *Helicobacter* and *Candida* that correlate with disease severity (11, 12). Despite these advances, current probiotic strategies remain largely empirical, constrained by an incomplete understanding of the bioactive components mediating therapeutic effects. For instance, Prevotella-mediated exacerbation of psoriatic pathology via aberrant fatty acid metabolism can be reversed by antibiotic intervention (8), while probiotic supplementation with *Streptococcus thermophilus* or *Lactobacillus sakei* derivatives improves epidermal barrier function and attenuates psoriasis inflammation (13, 14). Notably, *Lactobacillus* spp. are the most commonly used probiotics in clinical trials for psoriasis treatment (15). However, merely focusing on probiotic therapies would fall short of addressing the pivotal aspects of microbiota-host interactions. Identifying the crucial components or bioactive molecules underlying the therapeutic effects of probiotics is an urgent priority that warrants intensive scientific investigation.

Emerging consensus posits that probiotic efficacy arises from a constellation of bacterial metabolites, extracellular vesicles (EVs), cell wall components, and secreted biomolecules (16). While EVs have garnered significant attention for their immunomodulatory roles (17, 18), there remains a scarcity of studies that compare the similarities and differences between various bioactive constituents derived from the same bacterium. This limitation not only hampers a comprehensive understanding of the advantages offered by EVs but also restricts the potential recognition of other bioactive constituents.

In this study, we identified a decreased abundance of *Lactobacillus* species as a conserved microbial signature among four inflammatory skin diseases, including psoriasis. Using *Lactobacillus plantarum* as a model, we isolated four bioactive fractions: cytoplasmic membrane vesicles (CMVs), bacterial lysate supernatant (BL-S), bacterial lysate precipitate (BL-P) and cell-free fermentation supernatant (CFS) to dissect their immunoregulatory properties. While CMVs and BL-S possess therapeutic potential, as evidenced by their anti-inflammatory, antioxidant, and anti-hyperkeratotic properties, CMVs uniquely suppressed pro-inflammatory M1 macrophages polarization, a hallmark of psoriatic immunopathology. In a murine psoriasis model, CMVs outperformed other fractions in suppressing inflammation and improving psoriasis symptoms. Our work not only establishes CMVs as Lp’s dominant immunoregulatory component but also provides a comparative framework for evaluating microbial-derived therapeutics, advancing the development of targeted, non-living microbiota-based interventions for inflammatory diseases.

## 2 Results

### 2.1 Decreased abundance of *Lactobacillus* spp. is associated with psoriasis and other inflammatory dermatoses

**Figure 1:**
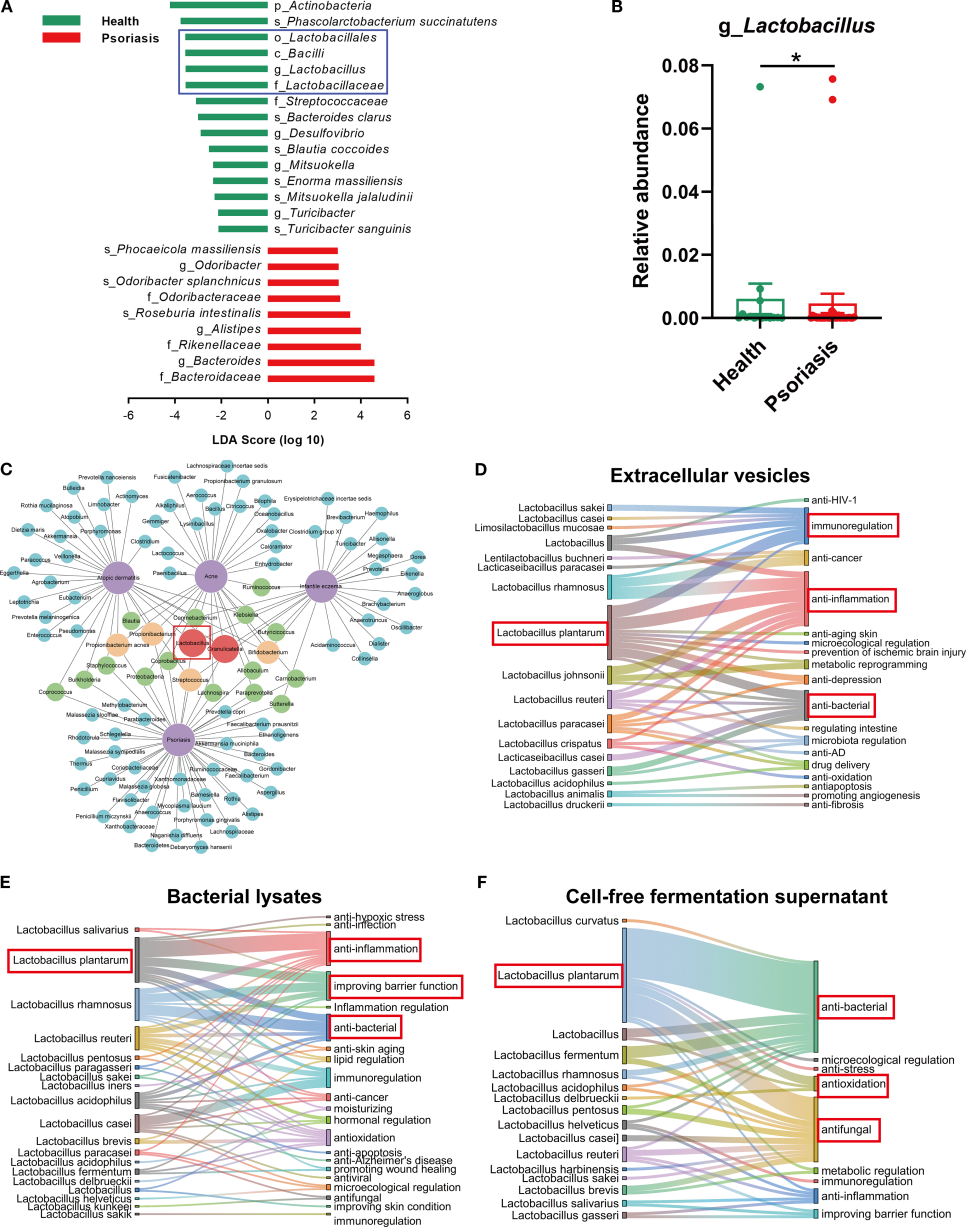
Reduced *Lactobacillus* abundance correlates with psoriasis and other inflammatory skin diseases. **(A)** Differential microbiome between healthy individuals and psoriasis patients based on LDA scores (-2 ≤ LDA ≤ 2; data derived from human metagenome sequencing of project PRJNA634145 in gutMDisorder online database). **(B)** Relative abundance of *Lactobacillus* in fecal samples from healthy individuals and psoriasis patients [the data source is the same as **(A)**]. **(C)** Network diagram showing the downregulated microbiome in four inflammatory skin diseases (atopic dermatitis, acne, infant eczema, and psoriasis, marked in purple). The linkages to diseases represent the decreased abundance trend of microorganisms in the corresponding diseases. Blue, green, orange, and red represent the microbiome downregulated in one, two, three, and four inflammatory skin diseases, respectively (data sourced from Disbiome online database). **(D–F)** Sankey diagram revealing the various therapeutic effects of different *Lactobacillus* extracellular vesicles, lysates or cell-free fermentation supernatant via bibliometric analysis. Data are presented as mean ± SD. Statistical significance was determined by the nonparametric Mann-Whitney U test (*p < 0.05) **(B)**. LDA, linear discriminant analysis.

To investigate the alterations in the gut microbiome of psoriasis patients, we compared the gut microbiota of healthy individuals with that of psoriasis patients using data from the gutMDisorder online database. The LEfSe analysis of the differential microbiota in psoriasis patients showed a notable increase in nine species and a significant decrease in fifteen species compared to the microbiota of healthy subjects (**Figure 1A**). Among these, *c_Bacilli*, *o_Lactobacillales*, *f_Lactobacteriaceae* and *g_Lactobacillus* were found to be less abundant in psoriasis patients in comparison to the healthy population (**Supplementary Figure S1**, **Figure 1B**). Following this, we conducted a detailed analysis within the Disbiome online database focusing on the microbiota of patients suffering from four distinct inflammatory skin diseases: atopic dermatitis, acne, infantile eczema and psoriasis. The analysis consistently demonstrated a decreased presence of *Lactobacillus* spp. across all these conditions (**Figure 1C**). The findings indicated that the diminished abundance of *Lactobacillus* spp. is a common feature among the microbiomes of various inflammatory skin diseases, suggesting that the loss of *Lactobacillus* spp. may play a significant role in the maintenance and exacerbation of these skin conditions. To identify specific *Lactobacillus* strains with therapeutic potential, we reviewed the literature on the therapeutic effects of extracellular vesicles (EVs), lysates, and cell-free supernatants (CFS) derived from this genus. *Lactobacillus plantarum* (*L. plantarum*, Lp) was determined to be the most therapeutically promising species. Lp EVs have been documented to exhibit immunomodulatory, anti-inflammatory, and antibacterial activities (**Figure 1D**); Lp lysates demonstrate anti-inflammatory effects, enhance skin barrier function, and possess antibacterial properties (**Figure 1E**); and Lp CFS displays antioxidant, antibacterial, and antifungal activities (**Figure 1F**).

### 2.2 Preparation and characterization of CMVs and bioactive constituents derived from *L. plantarum*

**Figure 2:**
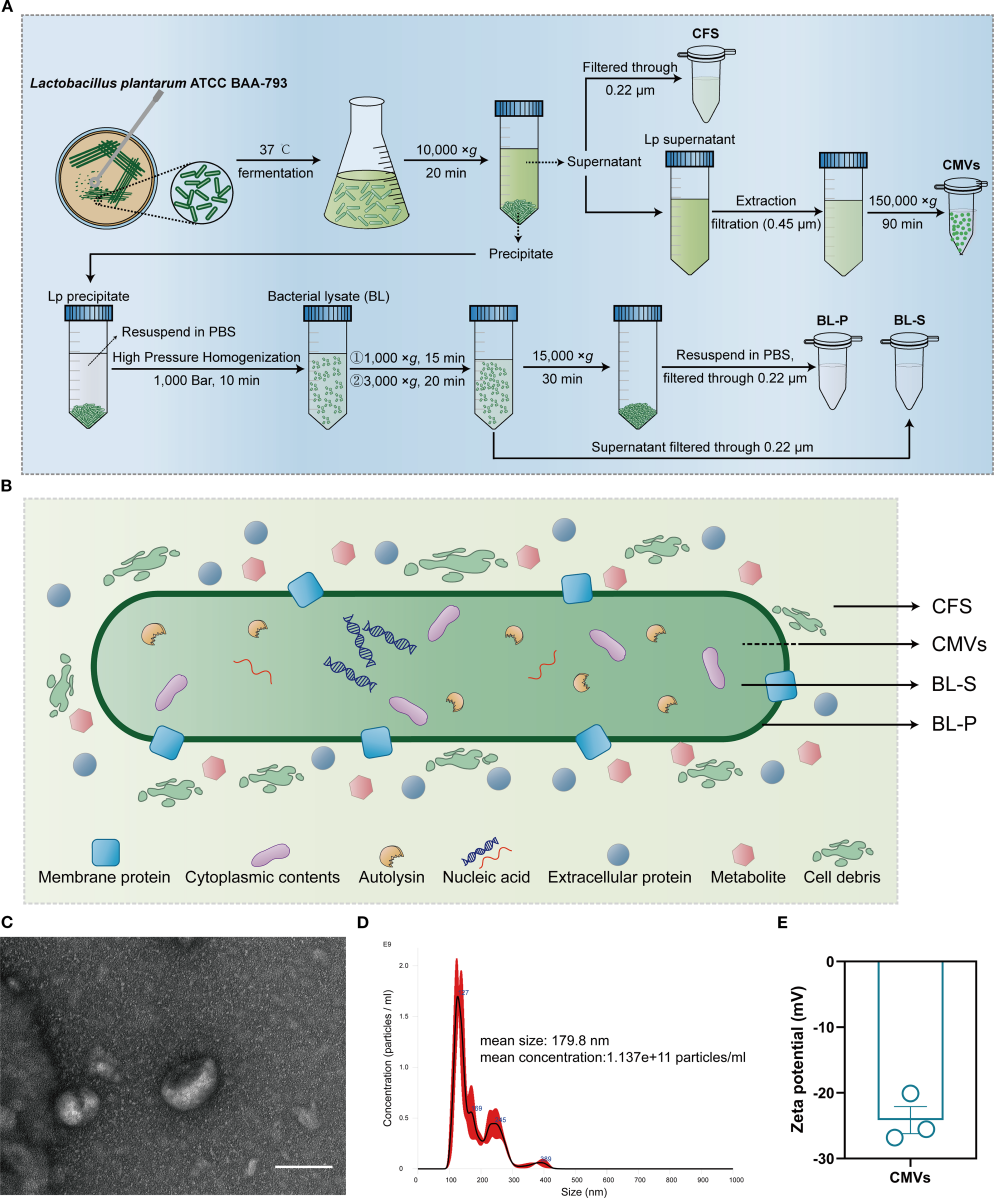
Extraction and characterization of CMVs and bioactive compounds from *L. plantarum*. **(A)** Flow diagram of the extraction and preparation of four bioactive components (CMVs, BL-S, BL-P and CFS) derived from *L. plantarum*. **(B)** Schematic plot of the sources and potential elements of the four bioactive components (CMVs, BL-S, BL-P and CFS). **(C)** Representative TEM image of CMVs. Scale bar: 100 nm. **(D)** Size distribution and concentration of CMVs as detected by NTA. **(E)** Typical zeta potential of CMVs (*n* = 3). Data are presented as mean ± SD. Lp, lactobacillus plantarum; CMVs, cytoplasmic membrane vesicles; BL-S, bacterial lysate supernatant; BL-P, bacterial lysate precipitate; CFS, cell-free fermentation supernatant.

Given the results presented, *L. plantarum* has been identified as the key candidate for developing further therapeutic approaches in this research. Following anaerobic fermentation culture, four bioactive components of *L. plantarum* ATCC BAA-793, namely CMVs, BL-S, BL-P and CFS, were extracted and prepared through distinct procedures (**Figure 2A**). The inclusions contained within the four bioactive components varied due to the differences in their extraction schemes: CMVs were secreted from intracellular sources, BL-S and BL-P were extracted from the supernatant and cell precipitate of bacterial lysate, respectively, and CFS was derived from the fermentation culture broth (**Figure 2B**). The discoidal bilayer membrane structure, a typical morphological hallmark of extracellular vesicles, was also observed in CMVs by transmission electron microscopy (TEM; **Figure 2C**). The nanoparticle tracking analysis (NTA) showed that the particle size of CMVs was evenly distributed around 179.8 nm, with a concentration of approximately 1.137e+11 particles/mL (**Figure 2D**). Additionally, dynamic light scattering (DLS) analysis revealed an average zeta potential of -24.14 mV for CMVs, indicating their good stability (**Figure 2E**).

### 2.3 Lp CMVs suppressed pro-inflammatory M1 polarization of macrophages

**Figure 3:**
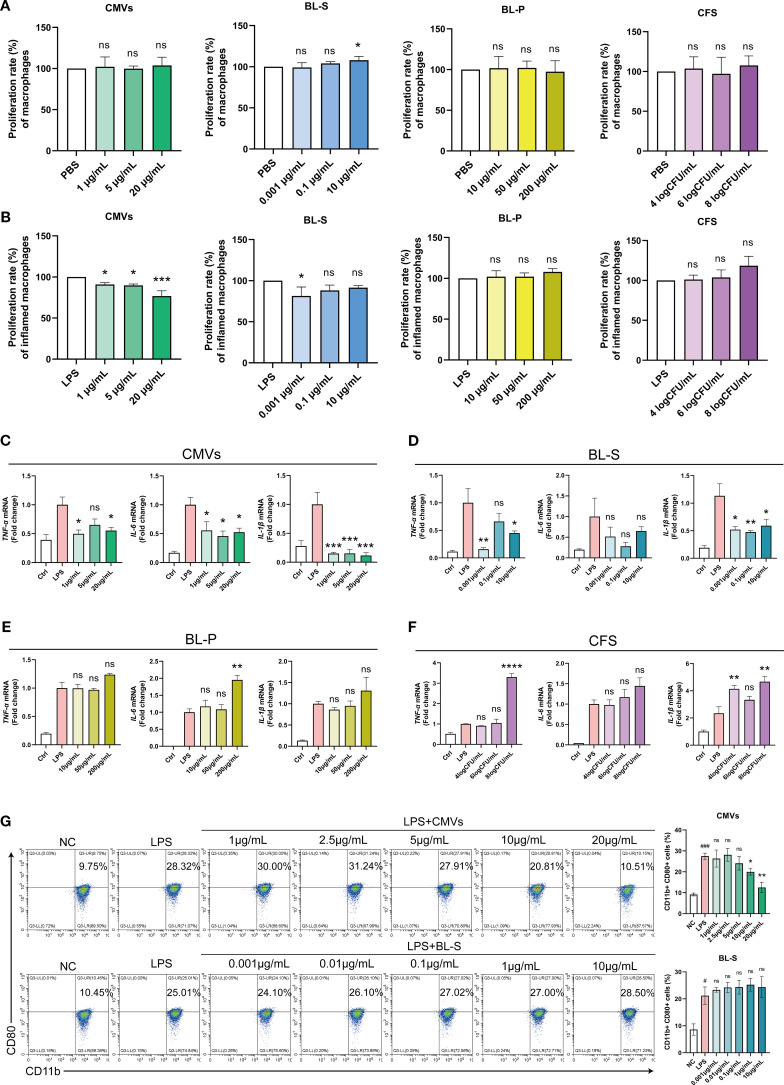
Lp CMVs inhibited macrophages M1 polarization. **(A)** The cellular activity of THP-1 macrophages treated with CMVs, BL-S, BL-P or CFS (*n* ≥ 4). **(B)** The cellular activity of inflammatory THP-1 macrophages treated with CMVs, BL-S, BL-P or CFS, with LPS induction (*n* = 3). **(C–F)** qPCR detection of mRNA expression of TNF-α, IL-6 and IL-1β in LPS-induced THP-1 cells co-treated with CMVs, BL-S, BL-P and CFS (*n* = 3). **(G)** Flow cytometry detection and quantitative analysis of the proportion of M1 (CD11b^+^ CD80^+^) macrophages in LPS-induced iBMDMs co-treated with CMVs and BL-S (*n* = 3). Data are presented as mean ± SD. Statistical significance was determined by one-way ANOVA followed by Dunnett’s *post hoc* test **(A–G)** for multiple comparisons (ns represents not significant, *p < 0.05, **p < 0.01, ***p < 0.001, ****p < 0.0001). CMVs, cytoplasmic membrane vesicles; BL-S, bacterial lysate supernatant; BL-P, bacterial lysate precipitate; CFS, cell-free fermentation supernatant; PBS, phosphate buffer saline; LPS, lipopolysaccharide; Ctrl, control; NC, negative control; TNF-α, tumor necrosis factor; IL-6, interleukin-6; IL-1β, interleukin-1β.

M1 phenotype macrophages are deemed to contribute to the escalation and maintenance of inflammatory responses by secreting pro-inflammatory mediators, involving in antigen presentation, and responding to oxidative stress (19). Concurrently, these actions may result in tissue injury and the progression towards chronic inflammation. As innate immune cells that massively infiltrate psoriatic skin lesion sites, macrophages activate adaptive immune responses and induce hyperproliferation and abnormal differentiation of keratinocytes by secreting various inflammatory cytokines (20–22). Hence, our initial exploration focused on assessing the impacts of bioactive constituents from *L. plantarum* on the inflammatory responses elicited by macrophages. In our initial investigation, we observed that among the four bioactive components, only BL-S at 10 μg/mL slightly enhanced the basal proliferation activity of PMA-induced THP-1 macrophages by 7.94% (*p* < 0.05), while the others had no significant effect (**Figure 3A**). In the LPS-induced macrophage proliferation model, CMVs significantly suppressed macrophage proliferation in a concentration-dependent manner, with inhibition rates reaching up to 23.21% (*p* < 0.0001) at the highest concentration (**Figure 3B**). BL-S exhibited inhibition only at the lowest concentration tested (0.001 μg/mL), reducing proliferation by 18.52% (*p* < 0.05). In contrast, BL-P and CFS showed no inhibitory effects on LPS-induced THP-1 macrophage proliferation. Following the differentiation of THP-1 cells into macrophages using PMA, we evaluated the inflammatory responses of these macrophages when exposed to four bioactive components from *L. plantarum*, under LPS-induced activation. Quantitative PCR analysis revealed that CMVs significantly suppressed the mRNA expression of pro-inflammatory cytokines in a concentration-dependent manner (**Figure 3C**). Specifically, CMVs treatment led to a marked reduction in TNF-α (up to 50.77%), IL-6 (up to 54.20%), and IL-1β (up to 88.44%; all *p* < 0.05 except one intermediate concentration of TNF-α). Similarly, BL-S also exhibited inhibitory effects on TNF-α and IL-1β expression across multiple concentrations, with reductions ranging from 33.79% to 84.32% (**Figure 3D**). Notably, the highest and lowest concentrations of BL-S significantly suppressed TNF-α (*p* < 0.05 and *p* < 0.01, respectively), while all three concentrations significantly reduced IL-1β expression (*p* < 0.05 or *p* < 0.01). In contrast, the effect of BL-S on the expression of IL-6 mRNA was not statistically significant. On the other hand, neither BL-P nor CFS showed any suppressive effect on these cytokines; instead, they either had no impact or even enhanced their expression (**Figures 3E, F**). The critical role of M1 macrophages in the persistent inflammation of psoriasis underscores the need to assess whether CMVs and BL-S, which have been confirmed to reduce the expression of inflammatory mediators, also exert a comparable impact on the polarization of macrophages. Flow cytometry analysis demonstrated that CMVs treatment resulted in a concentration-dependent reduction in the proportion of CD11b^+^CD80^+^ M1 pro-inflammatory macrophages in LPS-induced iBMDMs. Significant inhibition was observed at higher concentrations (≥ 10 μg/mL), with a maximum reduction of 54.45% (*p* < 0.01). In contrast, BL-S showed no significant effect on the polarization of M1 macrophages (**Figure 3G**). The presented data above indicate that among the four bioactive components from *L. plantarum*, CMVs stood out as the most effective in suppressing the secretion of inflammatory cytokines by macrophages and the polarization to the M1 pro-inflammatory phenotype. BL-S, on the other hand, displayed an effect that is second only to CMVs in reducing the production of inflammatory cytokines by macrophages.

### 2.4 Lp CMVs exerted antiproliferative and antioxidant effects in inflamed keratinocytes

**Figure 4:**
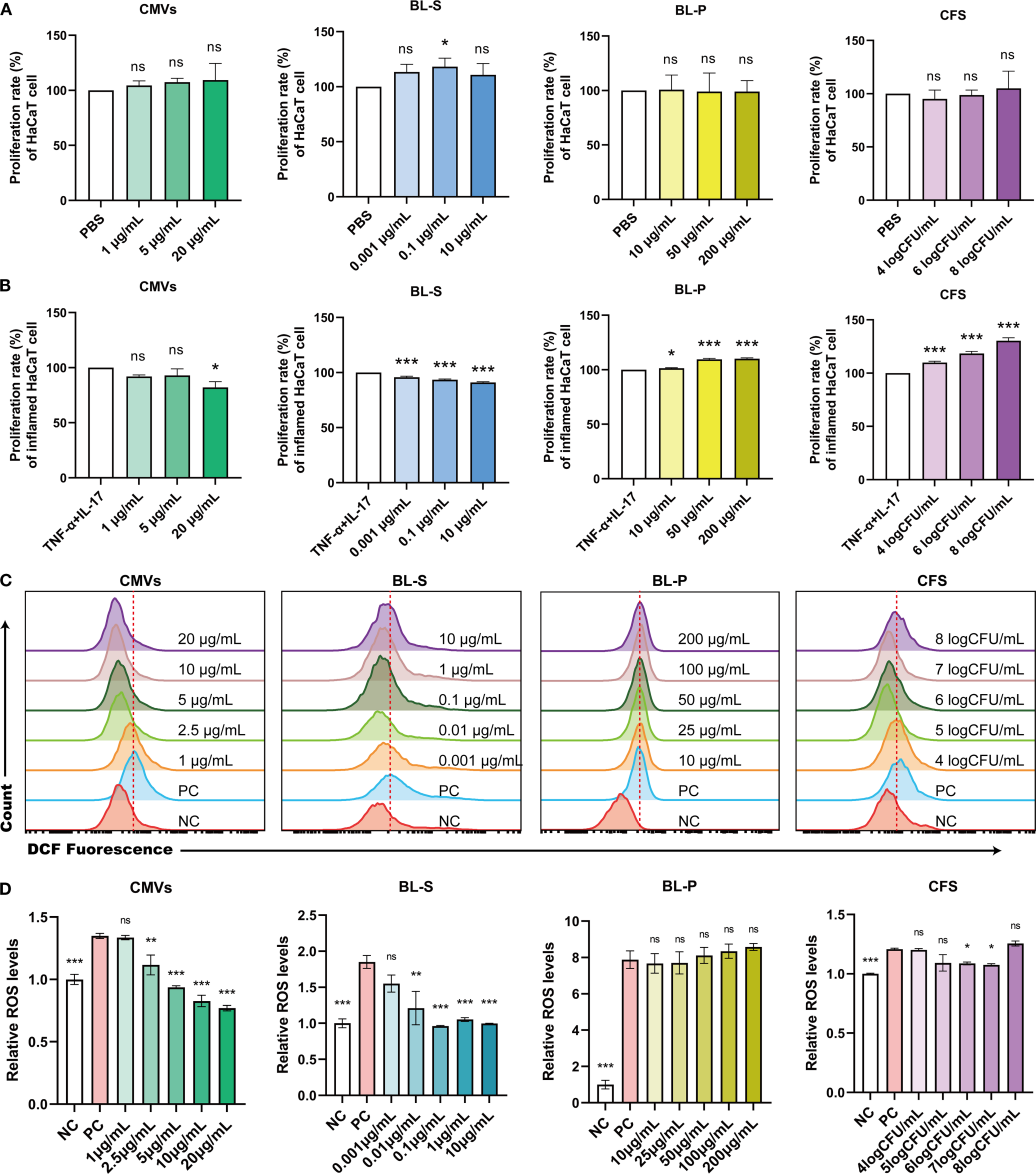
Lp CMVs had anti-proliferation and anti-oxidation effects on inflammatory keratinocytes. **(A)** The cellular activity of HaCaT cells treated with CMVs, BL-S, BL-P or CFS (*n* = 3). **(B)** The cellular activity of TNF-α/IL-17-induced HaCaT cells treated with CMVs, BL-S, BL-P or CFS (*n* = 3). **(C, D)** Flow cytometry was employed to detect and quantitatively analyze ROS levels in inflammatory HaCaT cells treated with CMVs, BL-S, BL-P or CFS (*n* = 3). Data are presented as mean ± SD. Statistical significance was determined by one-way ANOVA followed by Dunnett’s *post hoc* test **(A–D)** for multiple comparisons (ns represents not significant, *p < 0.05, **p < 0.01, ***p < 0.001, ****p < 0.0001). CMVs, cytoplasmic membrane vesicles; BL-S, bacterial lysate supernatant; BL-P, bacterial lysate precipitate; CFS, cell-free fermentation supernatant; PBS, phosphate buffer saline; TNF-α, tumor necrosis factor; IL-17, interleukin-17; NC, negative control; PC, positive control.

The pathogenesis of psoriasis is significantly exacerbated by a positive feedback loop between hyperkeratosis and oxidative stress driven by the increased production of reactive oxygen species (ROS), amplifying local inflammation and leading to a progressively worsening disorder (23, 24). In our initial investigation, we evaluated the potential cytotoxic effects of four bioactive components on keratinocyte cells. The findings indicated that under basal conditions, only BL-S at 0.1 μg/mL enhanced the proliferation of HaCaT cells by 18.23% (*p* < 0.05), and all four tested bioactive components exhibited no toxicity towards HaCaT cells and did not significantly impede their proliferative capabilities (**Figure 4A**). In the subsequent experimental stage, we replicated an inflammatory environment by introducing TNF-α and IL-17. This setup allowed us to further investigate the effects of the four bioactive components on the proliferative responses of keratinocytes to inflammatory cues, that is, evaluating their potential to modulate hyperkeratosis in the context of inflammation. Upon inflammatory stimulation with TNF-α and IL-17, CMVs significantly inhibited cell proliferation, with a maximum reduction of 17.76% (*p* < 0.05) at 20 μg/mL. BL-S also suppressed excessive cell proliferation at multiple concentrations, with a maximum inhibition rate of 8.98% (*p* < 0.001), albeit this effect was relatively modest. In contrast, both BL-P and CFS markedly promoted inflammatory proliferation in a concentration-dependent manner. BL-P enhanced proliferation by up to 10.13% (*p* < 0.001), and CFS exhibited even stronger pro-proliferative effects, with stimulation reaching 30.38% (*p* < 0.001) at the highest concentration (**Figure 4B**). In light of the well-documented link between ROS and inflammatory tissue damage in psoriasis, we proceeded to investigate the potential effects of the four bioactive components on the heightened ROS levels within an inflammatory context. Flow cytometry and quantitative analysis demonstrated that both CMVs and BL-S significantly attenuated ROS production in HaCaT cells under inflammatory stimulation in a concentration-dependent manner (**Figures 4C, D**). CMVs treatment resulted in a maximum reduction of ROS levels by up to 42.90% (*p* < 0.001), with significant effects observed at concentrations ≥ 2.5 μg/mL. Similarly, BL-S strongly inhibited ROS generation, with maximal inhibition reaching 48.01% (*p* < 0.001), and significance achieved at concentrations ≥ 0.01 μg/mL. In contrast, BL-P showed no significant suppressive effect on ROS levels. Although CFS at intermediate concentrations (6–7log CFU/mL) elicited a modest but significant inhibition (approximately 10%, *p* < 0.05), higher concentrations failed to suppress ROS and even slightly increased its production, indicating an overall limited effect. The cumulative evidence presented in this section firmly establishes the antiproliferative and antioxidant functionalities of CMVs and BL-S in modulating inflammatory keratinocyte responses, with CMVs demonstrating a greater overall efficacy.

### 2.5 Different metabolite profiles were observed between Lp CMVs and BL-S

**Figure 5:**
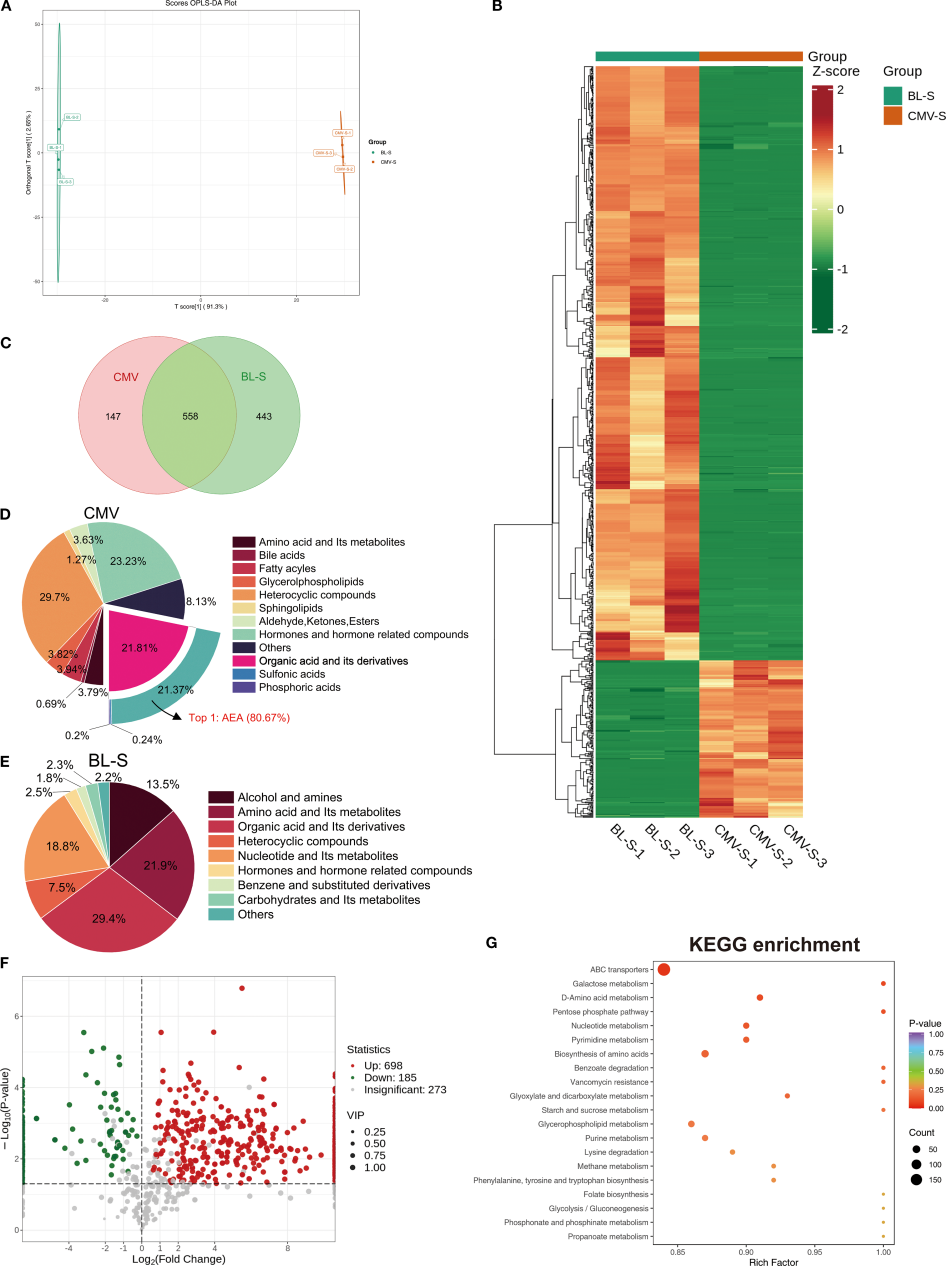
Lp CMVs and BL-S exhibited substantial variations in their metabolite composition patterns. **(A)** OPLS-DA score charts integrating both positive and negative ion modes in metabolomic analysis, feature green dots representing the BL-S samples and red dots indicating the CMVs samples. **(B)** Cluster heatmap analysis of metabolites in CMVs and BL-S. **(C)** Venn diagram of the metabolites contained in CMVs and BL-S. **(D, E)** The metabolites composition of CMVs and BL-S. **(F)** Volcano plot of differential metabolites between BL-S and CMVs. **(G)** KEGG enrichment analysis of differential metabolites (BL-S vs CMVs). CMVs, cytoplasmic membrane vesicles; BL-S, bacterial lysate supernatant.

To unravel the underlying reasons for the similarities and differences in the effects of CMVs and BL-S, we performed widely-targeted metabolomics analysis, aiming to investigate the material foundations of each. Orthogonal partial least squares discriminant analysis (OPLS-DA) and cluster heatmap analysis indicated significant differences in the metabolites between CMVs and BL-S (**Figures 5A, B**). The detailed metabolite profiling revealed the presence of 705 CMVs-derived metabolites and 1001 BL-S-derived metabolites, with an overlap of 558 metabolites common to both CMVs and BL-S (**Figure 5C**). The metabolites of CMVs predominantly consist of a diverse array of heterocyclic compounds, hormones and hormone-related compounds, and organic acid and its derivatives. Notably, anandamide (AEA) emerges as the most abundant organic acid derivative, comprising 80.67% of this category and accounting for 17.24% of the total metabolome (**Figure 5D**). In contrast, the metabolic landscape of BL-S is distinguished by a preponderance of organic acid and its derivatives, amino acid and its metabolites, in addition to alcohol and amines (**Figure 5E**). To further delineate the differential metabolites between CMVs and BL-S, a traceability analysis of metabolites utilizing the KEGG, HMDB, and CHEBI databases was conducted. This analysis revealed that, in comparison to CMVs, BL-S exhibited a significant upregulation of 698 metabolites and a notable downregulation of 185 metabolites (**Figure 5F**). Subsequently, KEGG pathway analysis for these differential metabolites was conducted. The distinct metabolites distinguishing CMVs from BL-S were predominantly concentrated in the ABC transporters, glycerophospholipid metabolism, biosynthesis of amino acids and purine metabolism (**Figure 5G**). In summary, widely-targeted metabolomics has uncovered significant differences in the metabolite profiles between CMVs and BL-S, suggesting that the variations in their efficacies may be attributed to these disparities.

### 2.6 AEA enriched in Lp CMVs inhibited cellular inflammation, oxidation and M1 macrophages polarization

**Figure 6:**
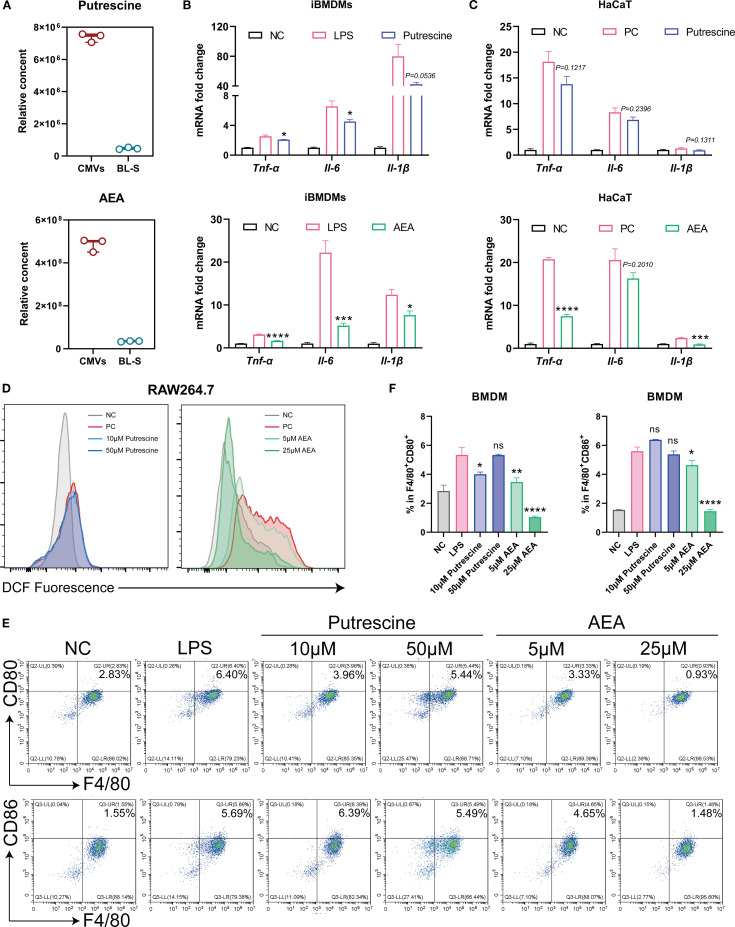
AEA, abundant in Lp CMVs, suppressed cellular inflammation, oxidation, and M1 macrophages polarization. **(A)** Relative content of Putrescine or AEA in CMV and BL-S by widely-targeted metabolomics (*n* = 3). **(B)** mRNA expression levels of *Tnf-α*, *Il-6*, and *Il-1β* in LPS-induced macrophages treated with Putrescine or AEA (*n* = 3). **(C)** mRNA expression levels of TNF-α, IL-6, and IL-1β in TNF-α and IL-17-induced HaCaT cells treated with putrescine or AEA (*n* = 3). **(D)** Flow cytometry analysis of intracellular ROS levels in inflammatory macrophages treated with putrescine or AEA (*n* = 3). **(E, F)** Representative flow cytometric images of the percentage of M1 (F4/80^+^ CD86^+^) macrophages and corresponding quantitative analysis (*n* = 3). Data are presented as mean ± SD. Statistical significance was determined by one-way ANOVA followed by Dunnett’s *post hoc* test (B, C, F) for multiple comparisons (ns represents not significant, *p < 0.05, **p < 0.01, ***p < 0.001, ****p < 0.0001). CMVs, cytoplasmic membrane vesicles; BL-S, bacterial lysate supernatant; NC, negative control; PC, positive control; LPS, lipopolysaccharide; AEA, anandamide; Tnf-α, tumor necrosis factor; Il-6, interleukin-6; Il-1β, interleukin-1β.

Putrescine and anandamide (AEA) are two primary metabolites that exhibit significant differences in concentrations between CMVs and BL-S. Putrescine is a nitrogenous aliphatic base widely present in living organisms, belonging to the polyamine class (25, 26). Studies have indicated that putrescine derived from symbiotic bacteria plays a role in regulating the balance of macrophages, manifested by increasing the proportion of anti-inflammatory macrophages and decreasing the proportion of pro-inflammatory macrophages (27). AEA, an endogenous, lipid-soluble cannabinoid, has been scientifically validated to exhibit a plethora of biological activities, including but not limited to anti-inflammatory, analgesic, neuroprotective, and anxiolytic effects (28–31). The content of putrescine and AEA in CMVs is about 15.68 times and 13.90 times of those in BL-S (**Figure 6A**). Given the substantial differences in the concentrations of these two metabolites between CMVs and BL-S, we conducted assessments of the anti-inflammatory, antioxidative and macrophage polarization-modulating effects of putrescine and AEA to identify the principal metabolites responsible for the observed efficacy in CMVs. qPCR outcomes revealed that both putrescine and AEA diminished the mRNA expression of pro-inflammatory cytokines (TNF-α, IL-6 and IL-1β) within inflamed macrophages. Notably, AEA demonstrated a more potent inhibitory impact on the inflammatory response in macrophages as compared to putrescine (**Figure 6B**). Specifically, in iBMDMs, AEA significantly reduced the expression of TNF-α by 47.18% (*p* < 0.001), IL-6 by 76.60% (*p* < 0.01), and IL-1β by 38.37% (*p* < 0.05), whereas putrescine showed a modest reduction of *IL-6* (31.15%, *p* < 0.05) and IL-1β (46.95%, *p* < 0.05) but no significant effect on TNF-α (17.60%, *p* = 0.0664). In the context of inflamed keratinocytes, putrescine failed to exhibit a significant anti-inflammatory effect, whereas AEA maintained its pronounced anti-inflammatory efficacy, markedly reducing TNF-α by 63.91% (*p* < 0.0001) and IL-1β by 62.75% (*p* < 0.001), despite a non-significant trend in IL-6 reduction (20.83%, *p* = 0.2010) (**Figure 6C**). Similarly, flow cytometry revealed that it is AEA, rather than putrescine, that significantly inhibited LPS-induced oxidative stress and decreased intracellular ROS levels in macrophages (**Figure 6D**). Subsequently, during a 7-day differentiation process of bone marrow-derived macrophages (BMDMs), either putrescine or AEA was administered, with LPS stimulation administered on the final day. Flow cytometric analysis was then performed to assess the macrophage polarization phenotypes upon completion of the induction. The results indicated that AEA significantly and concentration-dependently inhibited macrophage polarization toward the M1 phenotype (**Figures 6E, F**). At 25 μM, AEA markedly reduced the proportions of F4/80^+^CD80^+^ and F4/80^+^CD86^+^ cells by 80.51% and 73.96%, respectively (both *p* < 0.001). In contrast, putrescine exhibited only a limited and inconsistent effect: a slight reduction in F4/80^+^CD80^+^ cells was observed at 10 μM (24.86%, *p* < 0.05), while no significant changes were detected in F4/80^+^CD86^+^ cells at this concentration, or in either marker at 50 μM. Thus, it is evident that AEA serves as the key metabolite responsible for the predominant anti-inflammatory and antioxidative functions in CMVs, acting synergistically with other bioactive molecules to enhance immunosuppressive activities. Furthermore, the observed variations in efficacy between CMVs and BL-S are also highly likely to be attributed to the significant contributions of AEA.

### 2.7 Lp CMVs mitigated IMQ-induced psoriasis

**Figure 7:**
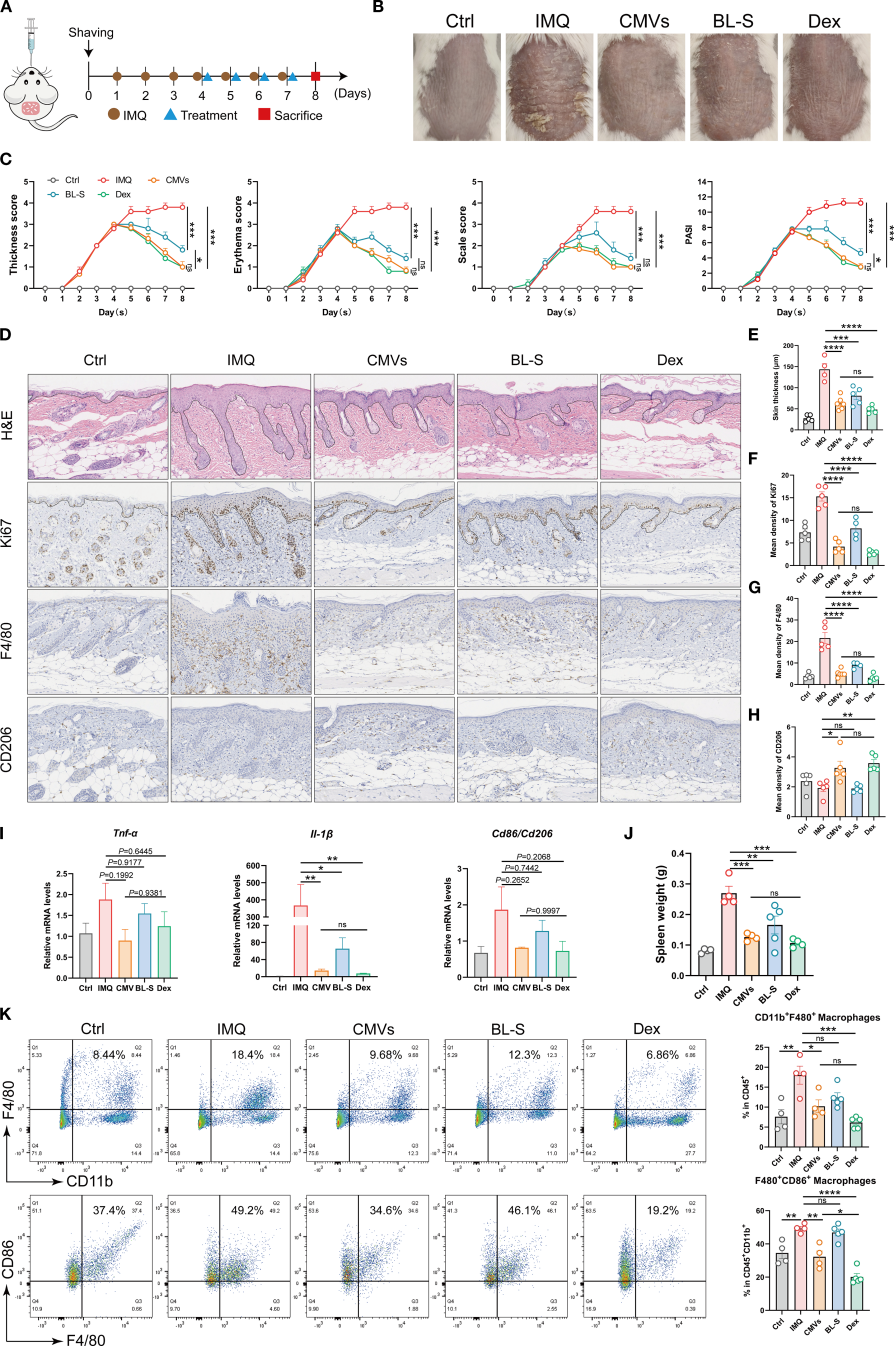
Lp CMVs alleviated IMQ-induced psoriasis. **(A)** Diagrammatic drawing of the oral drug administration schedule for day 4, 5, 6 and 7, coinciding with the daily regimen of IMQ application. **(B)** Representative images of mouse dorsal skin of each group on day 8. **(C)** Thickness scores, erythema scores, scale score and PASI scores of mice in each group were meticulously documented throughout the 8-day experiment (*n* = 5). **(D)** H&E staining, and Ki67, F4/80 and CD206 immunohistochemistry of lesional skins on day 8. Scale bar: 100 μm. **(E)** Quantitative analysis of skin H&E staining results in **(D)** (*n* ≥ 4). **(F–H)** Quantitative immunohistochemical analysis of skin Ki67, F4/80 and CD206 in **(D)** (*n* ≥ 4). **(I)** mRNA expression of TNF-α, IL-1β and CD86/CD206 in the mouse skin tissues on day 8 (*n* = 3). **(J)** Spleen weight of mice in each group over 8 days of the experiment (*n* = 5). **(K)** Representative flow cytometry plots and quantitative analysis of CD11b^+^F4/80^+^ macrophage cells (gated on positive CD45^+^ cells) and F4/80^+^ CD86^+^ macrophage cells (gated on positive CD45^+^CD11b^+^ cells, *n* ≥ 4). Data are presented as mean ± SD. Statistical significance was determined by one-way ANOVA followed by Tukey’s *post hoc* test **(C, E–K)** for multiple comparisons (ns represents not significant, *p < 0.05, **p < 0.01, ***p < 0.001, ****p < 0.0001). Ctrl, control; IMQ, imiquimod; CMVs, cytoplasmic membrane vesicles; BL-S, bacterial lysate supernatant; Dex, dexamethasone; Tnf-α, tumor necrosis factor; Il-6, interleukin-6; Il-1β, interleukin-1β.

Upon confirming the inflammatory and immunomodulatory functions of CMVs *in vitro*, it is essential to conduct comprehensive *in vivo* studies to elucidate its mechanisms of action and potential therapeutic applications. Imiquimod (IMQ) was utilized to induce a psoriasis-like murine model, with CMVs and BL-S administered orally for therapeutic intervention (**Figure 7A**). After treatment, the psoriasis symptoms in the CMVs group, including skin thickness, erythema and scaling, exhibited marked alleviation and the PASI significantly decreased. Quantitative analysis revealed that CMVs reduced thickness, erythema, scaling, and overall PASI scores by approximately 73-78% compared to the IMQ group (all *p* < 0.001), matching the effect of the positive control (the Dex group) without significant difference between the two treatments. Additionally, the BL-S group also showed moderate improvement across these parameters (reductions of 52-63%, all *p* < 0.001), though its effects were less pronounced than the CMVs group (**Figures 7B, C**). Upon induction of the psoriasis model via IMQ, the body weight of mice significantly decreased compared to normal mice (Ctrl group). However, there were no significant differences in body weight changes among the treatment groups when compared to the IMQ group (**Supplementary Figure S2**). Furthermore, H&E staining analysis of skin tissue revealed that CMVs and BL-S treatments significantly reduced skinfold thickness by inhibiting epidermal hyperproliferation. Compared with the IMQ group, CMVs administration resulted in a 56.11% decrease in skin thickness. This efficacy was comparable to that of the positive control Dex, which reduced skin thickness by 66.42% (**Figures 7D–F**). Immunohistochemical analysis results indicated that both CMVs and BL-S treatments suppressed F4/80^+^ cell infiltration in the lesional skin, with CMVs reducing F4/80^+^ density by 76.01%, a result again comparable to Dex (85.45% reduction). Notably, only CMVs and Dex promoted an increase in CD206^+^ cells (69.96% and 86.46%, respectively), with no significant different between the two groups (**Figures 7D, G, H**). qPCR analysis revealed a trend towards decreased mRNA levels of the skin inflammatory cytokines *TNF-α* and *IL-1β*, as well as the M1/M2 macrophage phenotype markers *CD86*/*CD206*, following CMVs treatment. Notably, CMVs led to a significant 96.07% decrease in skin *IL-1β* (**Figure 7I**). Simultaneously, we observed the relief of splenomegaly following treatment with CMVs, BL-S and Dex (**Figure 7J**), indicating the mitigation of the systemic inflammatory response. In particular, CMVs substantially reduced spleen weight by 53.29%, a result comparable to the 60.59% reduction achieved by Dex. As anticipated, flow cytometric analysis of the harvested murine spleens revealed that CMVs treatment significantly reduced the proportions of splenic CD11b^+^ F4/80^+^ macrophages (by 43.34%) and F4/80^+^ CD86^+^ pro-inflammatory macrophages (by 34.63%). Although effective, the reduction in F4/80^+^ CD86^+^ macrophages by CMVs was significantly less pronounced than that achieved by Dex (59.19% reduction) (**Figure 7K**).

Finally, we assessed the *in vivo* biocompatibility. Histological images of the heart, liver, spleen, lung and kidney revealed no significant alterations in tissue structure and cellular morphology across all groups, indicating the biocompatibility of CMVs and BL-S derived from *L. plantarum* (**Supplementary Figure S3**).

## 3 Discussion

Host-microbial interactions play a pivotal role in maintaining physiological homeostasis, with the gut microbiota-immune axis emerging as a critical regulator of chronic inflammatory disorders. Accumulating evidence suggests that commensal microbiota orchestrate immune responses through innate immune conditioning, barrier integrity maintenance, and modulation of inflammatory cascades, mediated by microbial metabolites such as short-chain fatty acids, secondary bile acids, lactic acid, and bacteriocins (32).

Notably, the systemic inflammation and immune dysregulation characteristic of psoriasis are strongly associated with microbial dysbiosis. Our data analysis revealed a marked depletion of Lactobacillus — a prototypical probiotic genus — across inflammatory skin diseases (**Figure 1**). This microbial imbalance may compromise intestinal barrier function, facilitating bacterial translocation and subsequent systemic immune perturbations (33), thereby underscoring the therapeutic rationale for probiotic interventions.

In this study, we systematically investigated the functional heterogeneity of bioactive components derived from Lp, including CMVs, BL-S, BL-P, and CFS. Both CMVs and BL-S demonstrated significant anti-inflammatory, antioxidant, and antiproliferative effects *in vitro* (**Figures 2**-**4**). However, a notable difference in efficacy was observed between CMVs and BL-S. CMVs significantly inhibited the polarization of macrophages towards the M1 phenotype (**Figure 3F**), whereas BL-S likely modulates inflammation through alternative pathways, potentially involving, potentially involving non-specific regulation of inflammatory mediators or metabolic reprogramming. Metabolomic profiling identified AEA, a signaling lipid derivative as a key bioactive metabolite enriched in both CMVs and BL-S, as disclosed by metabolomics, and has been validated to exert anti-inflammatory, antioxidant, and macrophage phenotype-regulatory properties (**Figure 6**). However, the markedly lower AEA concentration in BL-S compared with CMVs may be related to the lower anti-inflammatory activity of BL-S compared with CMVs and the lack of capacity to regulate macrophage polarization. However, the moderate anti-inflammatory and antioxidant effects observed with BL-S may be attributed not only to its residual AEA content but also to its distinct metabolomic profile—particularly its abundance of functional amino acids such as L-proline (20.45 times that of CMVs) and L-lysine (97.78 times that of CMVs). Studies have reported that proline supplementation can help alleviate cellular inflammation and ROS levels (34), acting as a scavenger to protect cells from oxidative damage (35). Likewise, lysine and its polymeric derivatives have been documented to exhibit anti-inflammatory and antioxidant activities (36, 37; 38). These findings suggest that both AEA and additional metabolites may contribute to the pharmacological benefits of BL-S. Intriguingly, although the level of putrescine which has been previously established to modulate the balance of intestinal macrophages (27) were elevated in CMVs, its contribution to M1 macrophage suppression appeared negligible (**Figure 6E**), suggesting the involvement of other synergistic components.

Existing research provides potential clues regarding the mechanisms of AEA. As an endogenous ligand naturally present in the endocannabinoid system, AEA acts as an intermediary between the gut microbiota and host metabolism in the body. Studies have demonstrated that the anti-inflammatory effects of gut probiotics are at least partially mediated by the endocannabinoid system, specifically through AEA, which facilitated the production of high levels of short-chain fatty acids like butyrate by gut probiotics to combat inflammation (39). Furthermore, previous studies have established that endocannabinoid signaling enhances gut barrier integrity and synergizes with microbial metabolites like butyrate to exert systemic anti-inflammatory effects (40, 41). In this study, consistent with the *in vitro* findings, CMVs exhibited superior therapeutic efficacy in a psoriasis model, significantly alleviating psoriatic inflammation and reducing proinflammatory macrophage infiltration compared to BL-S (**Figure 7**). This functional superiority likely stems from CMVs’ unique metabolomic signature, particularly AEA-mediated activation of the endocannabinoid system.

**Figure 8:**
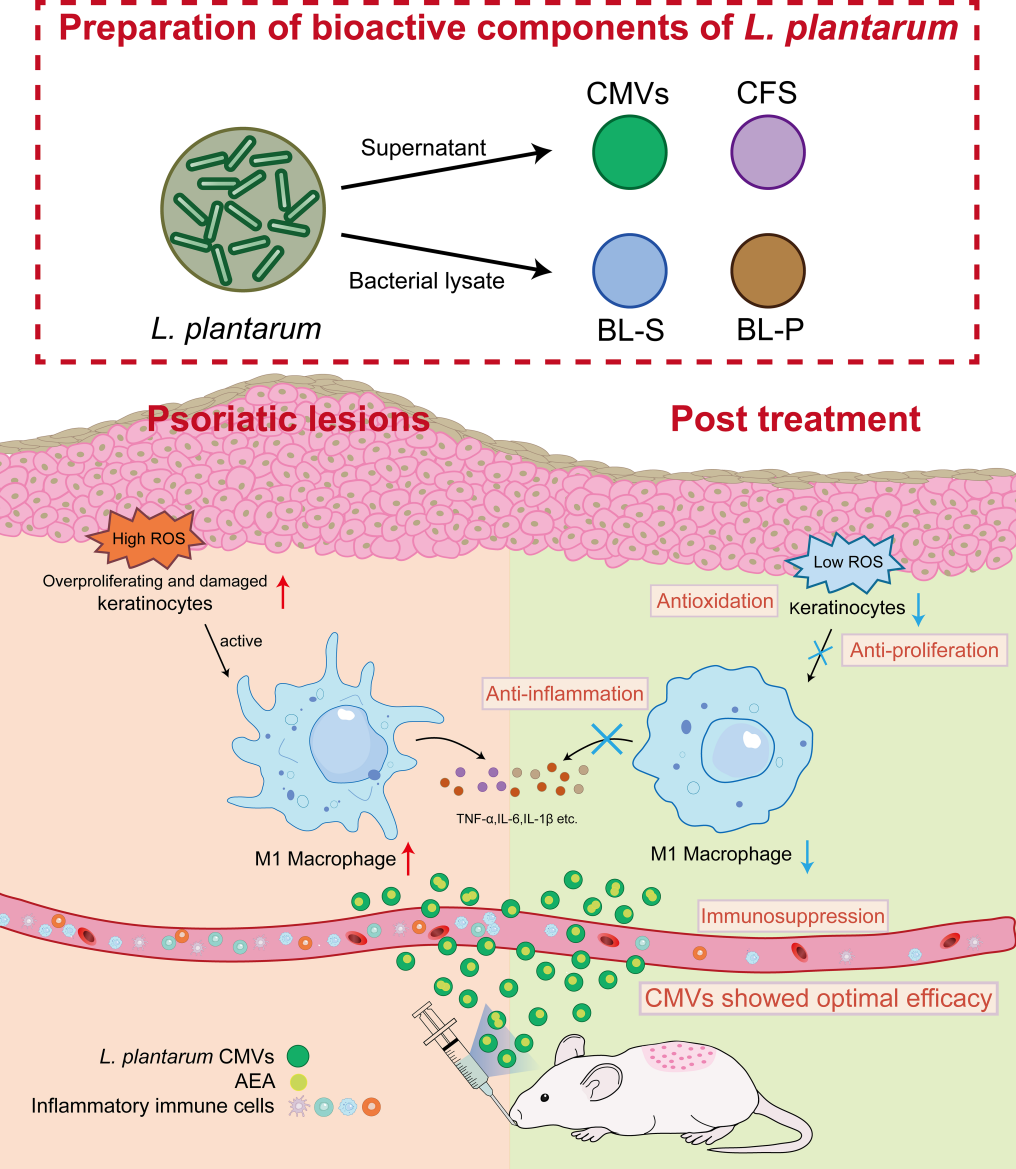
Schematic illustration of *L. plantarum* CMVs inhibiting excessive proliferation and ROS of keratinocytes, and suppressing inflammatory macrophages to alleviate symptoms of psoriasis in mice. *L. plantarum, lactobacillus plantarum;* CMVs, cytoplasmic membrane vesicles; BL-S, bacterial lysate supernatant; BL-P, bacterial lysate precipitate; CFS, cell-free fermentation supernatant; AEA, anandamide; ROS, reactive oxygen species; TNF-α, tumor necrosis factor; IL-6, interleukin-6; IL-1β, interleukin-1β.

In summary, based on these findings, we propose a “metabolome-immune crosstalk” model: four bioactive constituents, CMVs, BL-S, BL-P, and CFS derived from *L. plantarum*, exhibited distinct functional roles, which may be related to their diverse compositions of metabolites or other ingredients. Among them, *L. plantarum*-derived CMVs, enriched with AEA, demonstrated the best therapeutic efficacy in mitigating psoriatic pathogenesis through multi-dimensional regulation of macrophage polarization, keratinocyte hyerproliferation and oxidative stress (**Figure 8**). This highlights the therapeutic potential of strain-specific microbial metabolites and underscores the functional heterogeneity among probiotic components—even within the same species. In light of these findings, we anticipate future research to delve deeper into the specific mechanisms of AEA signaling networks and identification of BL-S constituents contributing to its anti-inflammatory effects. Translating these insights into clinical applications would offer new strategies for managing chronic inflammatory disorders like psoriasis.

## 4 Materials and methods

### 4.1 Bacterial strain

*Lactobacillus plantarum* ATCC BAA-793 was obtained from the strain repository of the laboratory. Strains were cultured in broth MRS medium (HKM) in anaerobic culture bags (MGC). All media, glass and plastic utensils used in the study were exposed to anaerobic conditions for at least 12 hours before use.

### 4.2 Cell culture

THP-1 cells (human monocytic cell line), purchased from American Type Culture Collection (ATCC), were cultured in RPMI 1640 medium (Gibco) supplemented with 10% fetal bovine serum (FBS, ExCell Bio) and 1% penicillin-streptomycin (P/S, Gibco), and induced into mononuclear macrophages by 50ng/mL phorbol-12-myristate-13-acetate (PMA, Sigma-Aldrich) for 2 days. iBMDMs (immortalized bone marrow-derived macrophages), RAW264.7 cells (mouse mononuclear macrophage cell line), HaCaT cells (human keratinocyte cell line), and L929 cells (mouse fibroblast cells line) were purchased from ATCC and cultured in Dulbecco’s Modified Eagle’s medium (DMEM, Gibco) supplemented with 10% FBS and 1% P/S. All cells were grown at 37 °C in a 5% CO_2_ atmosphere.

### 4.3 Extraction of BMDMs

BMDMs were extracted from the femurs and tibias of C57BL/6 mice (aged 6–8 weeks). The bones were washed with PBS containing 1% P/S and then flushed with a syringe filled with ice-cold medium to collect the bone marrow. The collected bone marrow fluid was centrifuged to pellet the bone marrow cells, which were then lysed with an appropriate volume of red blood cell lysis buffer (Solarbio) on ice for 2 minutes before another centrifugation to collect the cells. These cells were subsequently cultured in RPMI 1640 medium supplemented with 10% FBS, 1% P/S, and 30% supernatant from L929 cells for 7 days.

### 4.4 Data retrieval and microbiome analysis

The gutMDisorder online database was used to acquire the published human metagenome sequencing data PRJNA634145. The project employed shotgun metagenomic analysis in 15 healthy subjects and 33 psoriasis patients. To investigate the gut microbiome associated with psoriasis, LEfSe analysis and abundance statistics were employed to identify distinct microbiota between healthy individuals and psoriasis patients. Further confirmation of inflammation-associated microflora was analyzed in the Disbiome online database for bacterial abundance changes in four inflammatory skin diseases: atopic dermatitis, acne, infantile eczema and psoriasis.

### 4.5 Bibliometric and visualization analysis

Web of science Database serves as a professional data source for search queries. As depicted in **Figure 1D**, within the Web of Science Core Collection, the search was restricted to the Science Citation Index Expanded (SCI-EXPANDED) with the query focused on “lactobacillus vesicle [Topic]”, “lactobacillus lysate [Topic]” and “lactobacillus cell-free fermentation supernatant [Topic]”, yielding a total of 202, 205 and 157 citations, respectively. The inclusion criteria were set to include literatures related to the therapeutic functions of bacteria-derived constituents. All retrieved citations were preserved, then the therapeutic functions of bacterial-derived bioactive constituents studied in the literatures were analyzed and hot bacterium were visualized analyzed by Origin.

### 4.6 Extraction of Lp bioactive constituents

*Lactobacillus plantarum* was cultured in MRS broth medium (HKM) under anaerobic conditions at 37 °C for 24 hours. After centrifugation, the bacterial precipitate and fermentation supernatant were collected. CMVs were isolated from bacterial fermentation supernatant by sequential vacuum filtration through 0.45 μm pore size membrane filters (at least twice to ensure complete removal of large-sized impurities), followed by ultracentrifugation at 150,000 × *g* for 90 min. CFS was obtained by sterile filtration of the fermentation supernatant through 0.22 μm sterile membrane filters. The bacterial precipitate was washed with PBS, subjected to high-pressure homogenization for cell disruption, and then the cell debris and bacterial lytic supernatant were collected. The cell debris, after weighing, were dissolved in PBS to prepare BL-P. The bacterial lytic supernatant was centrifuged at 1,000 × *g* for 10 minutes, 3,000 × *g* for 15 minutes, and 15,000 × *g* for 30 minutes to obtain BL-S. All bioactive constituents should be sterile filtered through 0.22 μm sterile membrane filters after preparation, with BL-S undergoing a gradient extrusion through membranes of 0.8 μm, 0.45 μm, and 0.22 μm at least 10 times.

### 4.7 Characterization of Lp CMVs

Lp CMVs were dripped onto a 230-mesh formvar-carbon-coated copper grid (ZJKY, DJZCM-02-002), incubated for 10 minutes. And then the excess liquid was removed and the grid was washed with PBS. Subsequently, CMVs on the copper grid were stained with 2% uranyl acetate for 1 minute, washed with distilled water, and air-dried in the dark. Transmission electron microscopy (JEM-1400, 120 kV) was used to acquire the TEM images. The particle size distributions and particle concentration were determined by nanoparticle tracking analysis (NTA, NS300, Malvern Panalytical). The zeta potential of CMVs was quantified using a Zetasizer Pro system (Malvern Panalytical, UK), with three independent measurements performed in PBS buffer. BCA Protein Assay Kit (Beyotime, APT-P0012) was used to measure the protein concentration.

### 4.8 Cell viability assay

The Cell Counting Kit-8 (CCK-8, WAPExBI0) was used to assess cell viability. PMA-induced THP-1 cells and HaCaT cells were seeded into 96-well plates and incubated with CMVs, BL-S, BL-P and CFS in the presence or absence of LPS or TNF-α and IL-17 for 24 hours. After treatment, cells were incubated with 10% (v/v) CCK-8 reagent prepared in serum-free basal medium for about 1 h at 37 °C. The absorbance at 450 nm was measured by a microplate reader, and cell viability was calculated.

### 4.9 RNA isolation and quantitative real-time PCR

Total RNA was extracted from cells or skin tissues using the Trizol reagent (Vazyme Biotech). The RNA concentration was measured with a Nanodrop One (Thermo Fisher Scientific). Then RNA was reverse transcribed into cDNA using HiScript III RT SuperMix for qPCR (TransGen Biotech). Subsequently, real-time fluorescent qPCR was performed using 2 × SYBR Green qPCR Mix (TransGen Biotech) in a LightCycler 96 (Roche). The relative gene expression fold changes were calculated using the 2^-ΔΔCt^ method. Comprehensive details regarding the qPCR primers employed in all experiments can be found in **Supplementary Table S1**.

### 4.10 ROS level detection

2’,7’-Dichlorofluorescin diacetate probe (DCFH-DA, Sigma) was used to detect intracellular ROS level. Cells were stimulated with LPS and treated with CMVs, BL-S, BL-P or CFS for 24 hours. Subsequently, cells were incubated with DCFH-DA probe prepared in serum-free basic medium at 37 °C for 20 minutes in dark, followed by washes with PBS for 3 times to remove excess probe. Cells were detached with 0.25% trypsin-EDTA, collected, and then analyzed for intracellular ROS level by flow cytometry (Cytoflex, Beckman Coulter).

### 4.11 Flow cytometry analysis

To assess the phenotypes of macrophages *in vitro*, iBMDMs or BMDMs were stimulated with LPS and treated with drugs. ​​The cells were then​​ incubated with fluorescently labeled antibodies on ice for 15 minutes in the dark ​​and subjected to​​ flow cytometric analysis. For iBMDMs, after co-incubation with LPS and CMVs or BL-S for 24 hours, the polarization phenotypes were detected by FITC-conjugated anti-mouse CD11b antibody (Biolegend) and PerCP/Cyanine5.5-conjugated anti-mouse CD80 antibody (Biolegend). For BMDMs, cells were treated with Putrescine or AEA for 7 days. During this period, the culture medium was replaced with fresh drug-containing medium every 48 h. Following this treatment, cells were stimulated with LPS for 1 day, and the polarization phenotypes were assessed by APC-conjugated anti-mouse F4/80 antibody (Biolegend), FITC-conjugated anti-mouse CD80 antibody (Biolegend) and PE-conjugated anti-mouse CD86 antibody (Biolegend). To detect the phenotypes of macrophages *in vivo*, single-cell suspensions were obtained by grinding spleens of mice through 100 μm cell sieves and then treating with red blood cell lysis buffer on ice for 5 minutes. The harvested cells were incubated with PerCP/Cyanine5.5-conjugated anti-mouse CD45 antibody (Biolegend), PE-conjugated anti-mouse CD11b (Biolegend) antibody, FITC-conjugated anti-mouse F4/80 antibody (Biolegend), and APC-conjugated anti-mouse CD86 antibody (Biolegend) on ice for 20 minutes in dark before detection. After the staining process was complete, the excess antibodies were washed away, and the cell suspensions were subjected to flow cytometry.

### 4.12 Metabolomics analysis of Lp CMVs and BL-S

Metabolite composition of CMVs and BL-S was analyzed by LC-MS/MS: (1) Sample extraction: After thawing on ice, samples were mixed with 80% methanol-water containing internal standards and vortexed for 3 min. Following three cycles of liquid nitrogen freezing and thawing with vortexing, centrifugation was performed at 4 °C and 12,000 rpm for 10 min. The supernatant was dried and reconstituted in 70% methanol-water, then vortexed and sonicated in an ice-water bath. After final centrifugation (4 °C, 12,000 rpm, 3 min), the supernatant was transferred to vial inserts for analysis. (2) Chromatography-mass spectrometry: Analysis used UPLC (ExionLC AD) coupled with tandem mass spectrometry (QTRAP^®^). The T3 method employed a Waters ACQUITY UPLC HSS T3 C18 column (1.8 µm, 2.1 × 100 mm) with mobile phase A (0.1% formic acid in water) and B (0.1% formic acid in acetonitrile) at 0.4 mL/min. The gradient was: 0 min (95:5), 2.0 min (80:20), 5.0 min (40:60), 6.0-7.5 min (1:99), then return to 95:5 by 10.0 min (40 °C; 2 µL injection). MS conditions: ESI source at 500 °C; voltages ±4500 V; gas I 55 psi, gas II 60 psi; curtain gas 25 psi; high collision energy; MRM scanning with optimized declustering voltage and collision energy.

### 4.13 Induction and treatment of psoriasis in mouse models

Male BALB/c mice (SPF grade, aged 6–8 weeks, purchased from Zhuhai BestTest Bio-Thch Co., Ltd.) were adaptively fed for one week in a pathogen-free facility with ample diet before being used in experiments. The experimental protocol involving animal use was reviewed and approved by the Animal Ethics Committee of Sun Yat-sen University (Approval ID: SYSU-IACUC-2024-001484).

Mice were randomly divided into five groups and then carefully shaved using a depilatory cream. One day after depilation, 62.5 mg of 5% imiquimod (IMQ) cream (MedShine) was applied topically to the dorsal skin of mice in all groups except the healthy control group (Ctrl) once daily for 7 consecutive days to induce psoriasis-like symptoms. From days 4 to 7, CMVs or BL-S (both at 50μg per mouse per day in 100μL PBS) was administered via gavage, or dexamethasone (Dex) cream was applied topically as a positive control to mice in the corresponding groups, once daily. Mice were weighed daily. Disease severity was evaluated using the Psoriasis Area and Severity Index (PASI) by visual inspection of skin lesions. The assessment included three clinical indicators: thickness, erythema and scale. Each parameter was assigned a score ranging from 0 to 4, with 0 indicating non symptoms, 1 slight, 2 moderate, 3 severe, and 4 very severe. On the 8 day, mice were humanely sacrificed, and their skin, heart, liver, spleen, lungs and kidneys were harvested for subsequent experimentations.

### 4.14 Histology and immunohistochemistry analysis

The collected skin and other tissues were immersed in 4% paraformaldehyde overnight to fix the tissue, followed by paraffin embedding. Paraffin sections were stained with hematoxylin and eosin (H&E). For immunohistochemical analysis, the paraffin-embedded tissues were dewaxed and incubated with primary antibodies such as Ki67, F4/80, and CD206, followed by incubation with secondary antibodies and DAB staining. The tissue sections were observed and photographed under a 10 × magnifying lens.

### 4.15 Statistical analysis

All data were repeated in at least three independent biological experiments. Statistical analysis was performed using GraphPad Prism Ver 9.5.1, and data were presented as mean ± standard deviation (SD). Unpaired two-tailed Student’s *t* test or the nonparametric Mann-Whitney *U* test was used for comparison between two groups, and one-way analysis of variance (ANOVA) followed by Tukey’s or Dunnett’s *post hoc* test was used for comparison among multiple groups. Statistical significance levels were *p < 0.05, **p < 0.01, ***p < 0.001, ****p < 0.0001, and ns, not significant.

## Data Availability

The raw data supporting the conclusions of this article will be made available by the authors, without undue reservation.

## References

[1] Ansaldo E Farley TK Belkaid Y. Control of immunity by the microbiota. Annu Rev Immunol. (2021) 39:449–79. doi: 10.1146/annurev-immunol-093019-112348, PMID: 33902310

[2] Belkaid Y Harrison OJ. Homeostatic immunity and the microbiota. Immunity. (2017) 46:562–76. doi: 10.1016/j.immuni.2017.04.008, PMID: 28423337 PMC5604871

[3] Hou K Wu ZX Chen XY Wang JQ Zhang D Xiao C. Microbiota in health and diseases. Signal Transduct Target Ther. (2022) 7:135. doi: 10.1038/s41392-022-00974-4, PMID: 35461318 PMC9034083

[4] Zhang Y Wang H Sang Y Liu M Wang Q Yang H. Gut microbiota in health and disease: advances and future prospects. MedComm. (2020) 5:e70012. doi: 10.1002/mco2.70012, PMID: 39568773 PMC11577303

[5] Lowes MA Suárez-Fariñas M Krueger JG. Immunology of psoriasis. Annu Rev Immunol. (2014) 32:227–55. doi: 10.1146/annurev-immunol-032713-120225, PMID: 24655295 PMC4229247

[6] Uppala R Tsoi LC Harms PW Wang B Billi AC Maverakis E. Autoinflammatory psoriasis”-genetics and biology of pustular psoriasis. Cell Mol Immunol. (2021) 18:307–17. doi: 10.1038/s41423-020-0519-3, PMID: 32814870 PMC8027616

[7] Mahmud MR Akter S Tamanna SK Mazumder L Esti IZ Banerjee S. Impact of gut microbiome on skin health: gut-skin axis observed through the lenses of therapeutics and skin diseases. Gut Microbes. (2022) 14:2096995. doi: 10.1080/19490976.2022.2096995, PMID: 35866234 PMC9311318

[8] Zhao Q Yu J Zhou H Wang X Zhang C Hu J. Intestinal dysbiosis exacerbates the pathogenesis of psoriasis-like phenotype through changes in fatty acid metabolism. Signal Transduct Target Ther. (2023) 8:40. doi: 10.1038/s41392-022-01219-0, PMID: 36710269 PMC9884668

[9] Todberg T Egeberg A Zachariae C Sørensen N Pedersen O Skov L. Patients with psoriasis have a dysbiotic taxonomic and functional gut microbiota. Br J Dermatol. (2022) 187:89–98. doi: 10.1111/bjd.21245, PMID: 35289939

[10] Boix-Amorós A Badri MH Manasson J Blank RB Haberman RH Neimann AL. Alterations in the cutaneous microbiome of patients with psoriasis and psoriatic arthritis reveal similarities between non-lesional and lesional skin. Ann Rheum Dis. (2023) 82:507–14. doi: 10.1136/ard-2022-223389, PMID: 36600182 PMC11131958

[11] Olejniczak-Staruch I Ciążyńska M Sobolewska-Sztychny D Narbutt J Skibińska M Lesiak A. Alterations of the skin and gut microbiome in psoriasis and psoriatic arthritis. Int J Mol Sci. (2021) 22:3998. doi: 10.3390/ijms22083998, PMID: 33924414 PMC8069836

[12] Fyhrquist N Muirhead G Prast-Nielsen S Jeanmougin M Olah P Skoog T. Microbe-host interplay in atopic dermatitis and psoriasis. Nat Commun. (2019) 10:4703. doi: 10.1038/s41467-019-12253-y, PMID: 31619666 PMC6795799

[13] Di Marzio L Cinque B Cupelli F De Simone C Cifone MG Giuliani M. Increase of skin-ceramide levels in aged subjects following a short-term topical application of bacterial sphingomyelinase from. Streptococcus thermophilus. Int J Immunopathol Pharmacol. (2008) 21:137–43. doi: 10.1177/039463200802100115, PMID: 18336739

[14] Rather IA Bajpai VK Huh YS Han YK Bhat EA Lim J. Probiotic *Lactobacillus sakei* proBio-65 Extract Ameliorates the Severity of Imiquimod Induced Psoriasis-Like Skin Inflammation in a Mouse Model. Front Microbiol. (2018) 9:1021. doi: 10.3389/fmicb.2018.01021, PMID: 29867905 PMC5968580

[15] Rigon RB de Freitas ACP Bicas JL Cogo-Müller K Kurebayashi AK Magalhães RF. Skin microbiota as a therapeutic target for psoriasis treatment: Trends and perspectives. J Cosmet Dermatol. (2021) 20:1066–72. doi: 10.1111/jocd.13752, PMID: 32998180

[16] Cho YS Han K Xu J Moon JJ. Novel strategies for modulating the gut microbiome for cancer therapy. Adv Drug Delivery Rev. (2024) 210:115332. doi: 10.1016/j.addr.2024.115332, PMID: 38759702 PMC11268941

[17] Díaz-Garrido N Badia J Baldomà L. Microbiota-derived extracellular vesicles in interkingdom communication in the gut. J Extracell Vesicles. (2021) 10:e12161. doi: 10.1002/jev2.12161, PMID: 34738337 PMC8568775

[18] Li M Mao B Tang X Zhang Q Zhao J Chen W. Lactic acid bacteria derived extracellular vesicles: emerging bioactive nanoparticles in modulating host health. Gut Microbes. (2024) 16:2427311. doi: 10.1080/19490976.2024.2427311, PMID: 39538968 PMC11572086

[19] Chen S Saeed AFUH Liu Q Jiang Q Xu H Xiao GG. Macrophages in immunoregulation and therapeutics. Signal Transduct Target Ther. (2023) 8:207. doi: 10.1038/s41392-023-01452-1, PMID: 37211559 PMC10200802

[20] Xia T Fu S Yang R Yang K Lei W Yang Y. Advances in the study of macrophage polarization in inflammatory immune skin diseases. J Inflammation (Lond). (2023) 20:33. doi: 10.1186/s12950-023-00360-z, PMID: 37828492 PMC10568804

[21] Leite Dantas R Masemann D Schied T Bergmeier V Vogl T Loser K. Macrophage-mediated psoriasis can be suppressed by regulatory T lymphocytes. J Pathol. (2016) 240:366–77. doi: 10.1002/path.4786, PMID: 27555499

[22] Sa SM Valdez PA Wu J Jung K Zhong. F Hall L. The effects of IL-20 subfamily cytokines on reconstituted human epidermis suggest potential roles in cutaneous innate defense and pathogenic adaptive immunity in psoriasis. J Immunol. (2007) 178:2229–40. doi: 10.4049/jimmunol.178.4.2229, PMID: 17277128

[23] Pleńkowska J Gabig-Cimińska M Mozolewski P. Oxidative stress as an important contributor to the pathogenesis of psoriasis. Int J Mol Sci. (2020) 21:6206. doi: 10.3390/ijms21176206, PMID: 32867343 PMC7503883

[24] Zhou Q Mrowietz U Rostami-Yazdi M. Oxidative stress in the pathogenesis of psoriasis. Free Radic Biol Med. (2009) 47:891–905. doi: 10.1016/j.freeradbiomed.2009.06.033, PMID: 19577640

[25] Tabor CW Tabor H. Polyamines in microorganisms. Microbiol Rev. (1985) 49:81–99. doi: 10.1128/mr.49.1.81-99.1985, PMID: 3157043 PMC373019

[26] Miller-Fleming L Olin-Sandoval V Campbell K Ralser M. Remaining mysteries of molecular biology: the role of polyamines in the cell. J Mol Biol. (2015) 427:3389–406. doi: 10.1016/j.jmb.2015.06.020, PMID: 26156863

[27] Nakamura A Kurihara S Takahashi D Ohashi W Nakamura Y Kimura S. Symbiotic polyamine metabolism regulate sepithelial proliferation and macrophage differentiation in the colon. Nat Commun. (2021) 12(1)2105. doi: 10.1038/s41467-021-22212-1, PMID: 33833232 PMC8032791

[28] Cabral GA Toney DM Fischer-Stenger K Harrison MP Marciano-Cabral F. Anandamide inhibits macrophage-mediated killing of tumor necrosis factor-sensitive cells. Life Sci. (1995) 56:2065–72. doi: 10.1016/0024-3205(95)00190-h, PMID: 7776833

[29] Molina-Holgado F Molina-Holgado E Guaza C Rothwell NJ. Role of CB1 and CB2 receptors in the inhibitory effects of cannabinoids on lipopolysaccharide-induced nitric oxide release in astrocyte cultures. J Neurosci Res. (2002) 67:829–36. doi: 10.1038/s41467-021-22212-1, PMID: 11891798

[30] Eljaschewitsch E Witting A Mawrin C Lee T Schmidt PM Wolf S. The endocannabinoid anandamide protects neurons during CNS inflammation by induction of MKP-1 in microglial cells. Neuron. (2006) 49:67–79. doi: 10.1016/j.neuron.2005.11.027, PMID: 16387640

[31] Turcotte C Chouinard F Lefebvre JS Flamand N. Regulation of inflammation by cannabinoids, the endocannabinoids 2-arachidonoyl-glycerol and arachidonoyl-ethanolamide, and their metabolites. J Leukoc Biol. (2015) 97:1049–70. doi: 10.1189/jlb.3RU0115-021R, PMID: 25877930

[32] Yang W Cong Y. Gut microbiota-derived metabolites in the regulation of host immune responses and immune-related inflammatory diseases. Cell Mol Immunol. (2021) 18:866–77. doi: 10.1038/s41423-021-00661-4, PMID: 33707689 PMC8115644

[33] Fry L Baker BS Powles AV Fahlen A Engstrand L. Is chronic plaque psoriasis triggered by microbiota in the skin? Br J Dermatol. (2013) 169:47–52. doi: 10.1111/bjd.12322, PMID: 23521130

[34] Choudhury D Rong N Senthil Kumar HV Swedick S Samuel RZ Mehrotra P. Proline restores mitochondrial function and reverses aging hallmarks in senescent cells. Cell Rep. (2024) 43:113738. doi: 10.1016/j.celrep.2024.113738, PMID: 38354087 PMC13092368

[35] Krishnan N Dickman MB Becker DF. Proline modulates the intracellular redox environment and protects mammalian cells against oxidative stress. Free Radic Biol Med. (2008) 44:671–81. doi: 10.1016/j.freeradbiomed.2007.10.054, PMID: 18036351 PMC2268104

[36] Huang D Maulu S Ren M Liang H Ge X Ji K. Dietary Lysine Levels Improved Antioxidant Capacity and Immunity via the TOR and p38 MAPK Signaling Pathways in Grass Carp, Ctenopharyngodon idellus Fry. Front Immunol. (2021) 12:635015. doi: 10.3389/fimmu.2021.635015, PMID: 33717179 PMC7947207

[37] Mine Y Zhang H. Anti-inflammatory effects of poly-L-lysine in intestinal mucosal system mediated by calcium-sensing receptor activation. J Agric Food Chem. (2015) 63:10437–47. doi: 10.1021/acs.jafc.5b03812, PMID: 26588227

[38] Li P Han F Cao W Zhang G Li J Zhou J. Carbon quantum dots derived from lysine and arginine simultaneously scavenge bacteria and promote tissue repair. Appl Mater Today. (2020) 19:100601. doi: 10.1016/j.apmt.2020.100601

[39] Vijay A Kouraki A Gohir S Turnbull J Kelly A Chapman V. The anti-inflammatory effect of bacterial short chain fatty acids is partially mediated by endocannabinoids. Gut Microbes. (2021) 13:1997559. doi: 10.1080/19490976.2021.1997559, PMID: 34787065 PMC8604388

[40] Everard A Belzer C Geurts L Ouwerkerk JP Druart C Bindels LB. Cross-talk between *Akkermansia muciniphila* and intestinal epithelium controls diet-induced obesity. Proc Natl Acad Sci U.S.A. (2013) 110:9066–71. doi: 10.1073/pnas.1219451110, PMID: 23671105 PMC3670398

[41] Rousseaux C Thuru X Gelot A Barnich N Neut C Dubuquoy L. *Lactobacillus acidophilus* modulates intestinal pain and induces opioid and cannabinoid receptors. Nat Med. (2007) 13:35–7. doi: 10.1038/nm1521, PMID: 17159985

