# Supplementary material for: Lactobacillus plantarum-derived cytoplasmic membrane vesicles as novel anti-inflammatory nanotherapeutics for psoriasis management
Source: Front Immunol. 2025 Oct 8;16:1647466. doi: 10.3389/fimmu.2025.1647466 (PMC12540108; doi:10.3389/fimmu.2025.1647466)
Supplement: Supplementary file 1 [file DataSheet1.pdf]

Supporting Information

Lactobacillus plantarum-Derived Cytoplasmic Membrane Vesicles as Novel Anti-Inflammatory Nanotherapeutics for Psoriasis Management

Yuedong Xie[1, 2], Guowen Lv[2, 3], Dandan Su[2], Manchun Li[2], Quanle Xu[3], Hongbo Chen[2]*, Fang Cheng[2]*, Dongling Dai[1]*

[1]International Medical Center, Endoscopy Center and Gastroenterology Department Shenzhen Children's Hospital, Shenzhen 518036, China.

[2]School of Pharmaceutical Sciences (Shenzhen), Shenzhen Campus of Sun Yat-sen University, Shenzhen 518107, China.

[3]College of Life Sciences, Northwest A&F University, Yangling 712100, China.

*Corresponding authors. E-mails: Hongbo Chen, chenhb7@mail.sysu.edu.cn; Fang Cheng, chengf9@mail.sysu.edu.cn; Dongling Dai, daidong3529@sina.com.

Supplementary information includes three figures and one table:

Figure S1. The abundance of Lactobacillus genus and its family, order and class in patients with psoriasis decreased continuously (related to Figure 1).

Figure S2. The body weight of mice in each group on day 8 (related to Figure 7).

Figure S3. *In vivo* safety evaluation of CMVs and BL-S (related to Figure 7).

Table S1. The sequences of the qPCR primers (related to Materials and methods).

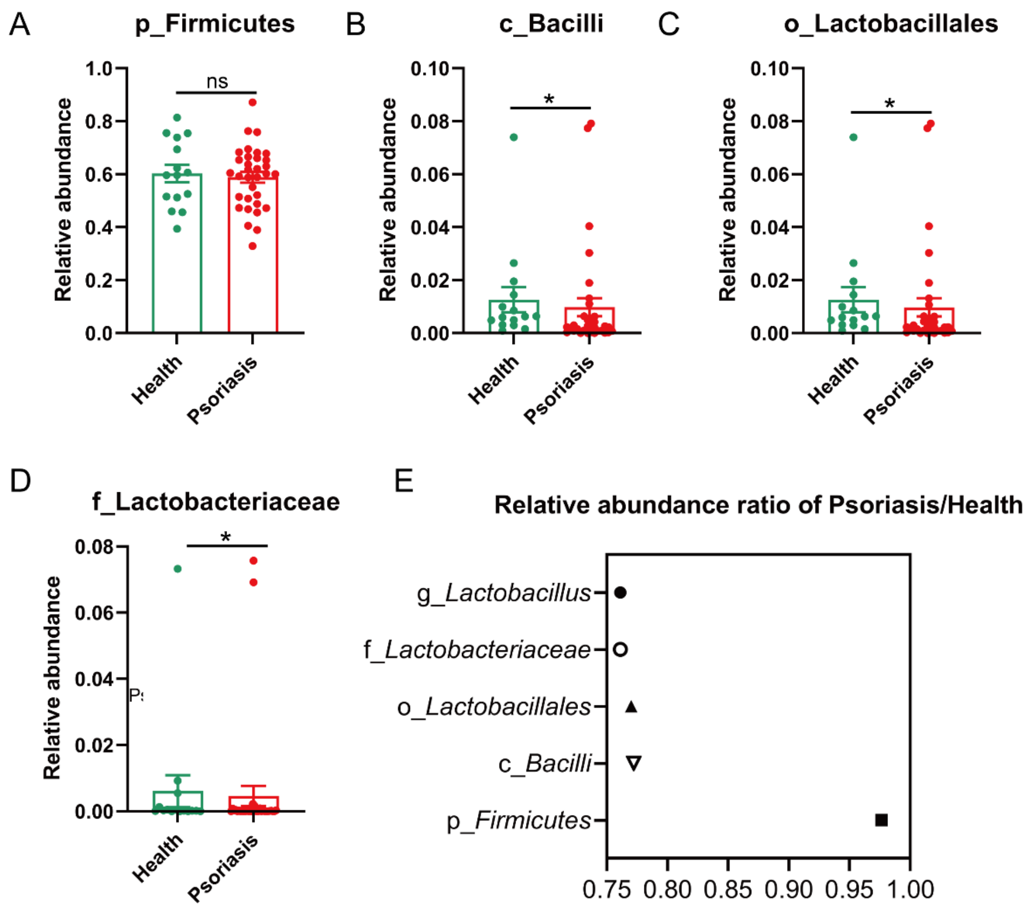

FIGURE S1. The abundance of Lactobacillus genus and its family, order and class in patients with psoriasis decreased continuously. **(A-D)** Relative abundance of *p_Firmicutes*, *c_Bacilli*, *o_Lactobacillales* and *f_Lactobacteriaceae* in fecal samples from healthy individuals and psoriasis patients (data derived from human metagenome sequencing of project PRJNA634145 in gutMDisorder online database). **(E)** The comparative ratio of the abundance of *p_Firmicutes*, *c_Bacilli*, *o_Lactobacillales*, *f_Lactobacteriaceae* and *g_Lactobacillus* between psoriasis patients and healthy individuals. Data are presented as mean ± SD. Statistical significance was determined by unpaired two-tailed *t* test (Figure S1A) or nonparametric Mann-Whitney *U* test (Figure S1B-D) (ns represents not significant, $^*p < 0.05$).

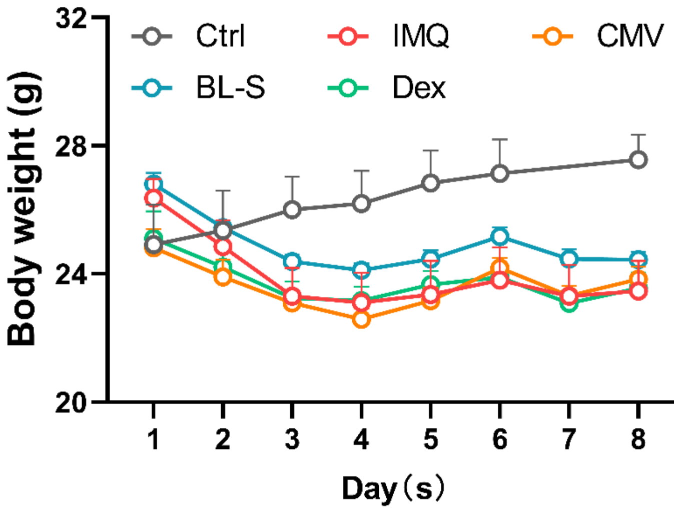

FIGURE S2. The body weight of mice in each group on day 8 (*n* = 5). Ctrl, control; IMQ, imiquimod; CMVs, cytoplasmic membrane vesicles; BL-S, bacterial lysate supernatant; Dex, dexamethasone.

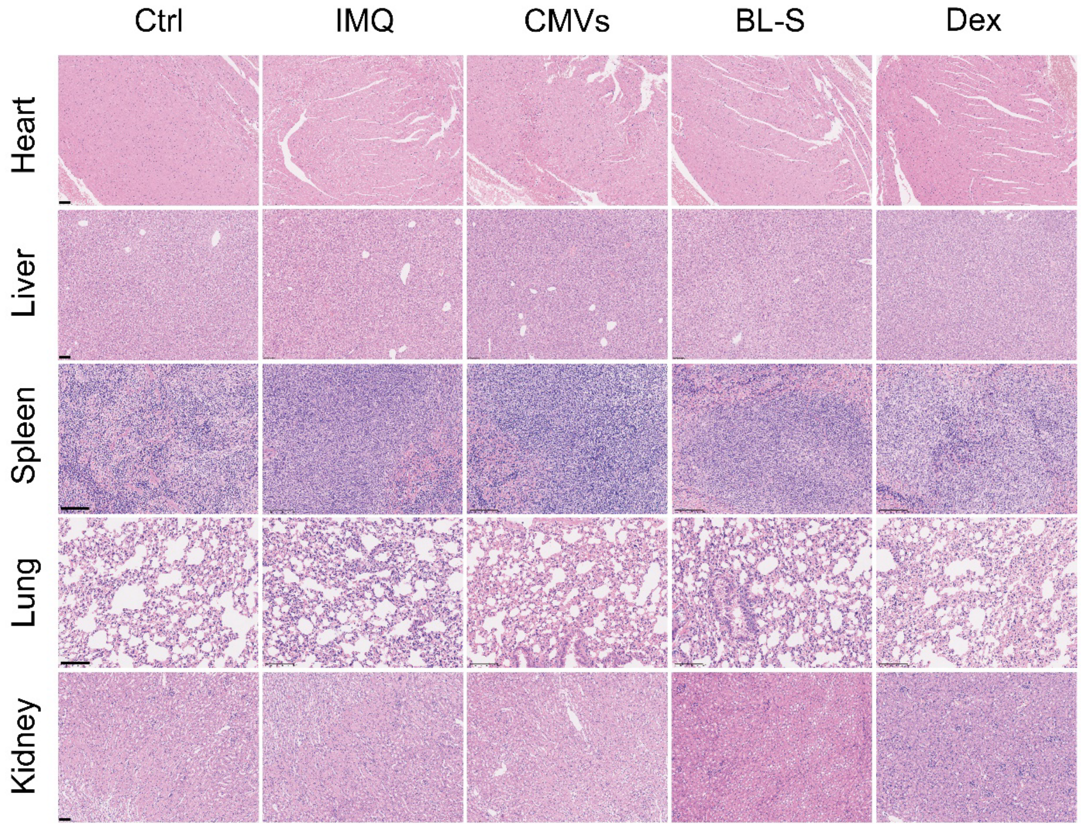

FIGURE S3. *In vivo* safety evaluation of CMVs and BL-S. Representative histological images of the heart, liver, spleen, lung, and kidney tissues from each group on day 8 (*n* = 5). Scale bar: 100 μm. Ctrl, control; IMQ, imiquimod; CMVs, cytoplasmic membrane vesicles; BL-S, bacterial lysate supernatant; Dex, dexamethasone.

Table S1. The sequences of the qPCR primers

Gene	Forward primer sequence 5'→3'	Reverse primer sequence 5'→3'
Human-*Gapdh*	TGTTGCCATCAATGACCCCTT	CTCCACGACGTACTCAGCG
Human-*Tnf-α*	GGACACCATGAGCACTGAAAGC	TGCCACGATCAGGAAGGAGAAG
Human-*Il-6*	AATTCGGTACATCCTCGACGGC	GCCAGTGCCTCTTTGCTGCTTT
Human-*Il-1β*	AAAGCTTGGTGATGTCTGGTC	GGACATGGAGAACACCACTTG
Mouse-*β-actin*	CACCCTGTGCTGCTCACCGA	AGTGTGGGTGACCCCGTCTCC
Mouse-*Tnf-α*	CTGAACTTCGGGGTGATCGG	GGCTTGTCACTCGAATTTTGAGA
Mouse-*Il-6*	GAGGATACCACTCCCAACAGACC	AAGTGCATCATCGTTGTTCATACA
Mouse-*Il-1β*	GAAATGCCACCTTTTGACAGTG	TGGATGCTCTCATCAGGACAG
Mouse-*Cd86*	TCTGCCGTGCCCATTTACAAAGG	TGCCCAAATAGTGCTCGTACAGAAC
Mouse-*Cd206*	AGACGAAATCCCTGCTACTG	CACCCATTCGAAGGCATTC
